# The Global Influenza Hospital Surveillance Network: A Multicountry Public Health Collaboration

**DOI:** 10.1111/irv.70091

**Published:** 2025-03-13

**Authors:** Marta C. Nunes, Cecile Chauvel, Sonia M. Raboni, F. Xavier López‐Labrador, Melissa K. Andrew, Nazish Badar, Vicky Baillie, Antonin Bal, Kedar Baral, Elsa Baumeister, Celina Boutros, Elena Burtseva, Daouda Coulibaly, Ben Cowling, Daria Danilenko, Ghassan Dbaibo, Gregory Destras, Ndongo Dia, Anca Cristina Drăgănescu, Heloisa I. G. Giamberardino, Doris Gomez‐Camargo, Laurence Josset, Parvaiz A. Koul, Jan Kyncl, Victor Alberto Laguna‐Torres, Odile Launay, Liem Binh Luong Nugyen, Shelly McNeil, Snežana Medić, Ainara Mira‐Iglesias, Alla Mironenko, Aneta Nitsch‐Osuch, Alejandro Orrico‐Sánchez, Nancy A. Otieno, Hadrien Regue, Guillermo M. Ruiz‐Palacios, Afif Ben Salah, Muhammad Salman, Oana Săndulescu, Viviana Simon, Anna Sominina, Emilia Sordillo, Mine Durusu Tanriover, Serhat Unal, Harm van Bakel, Philippe Vanhems, Tao Zhang, Catherine Commaille‐Chapus, Camille Hunsinger, Joseph Bresee, Bruno Lina, John W. McCauley, Justin R. Ortiz, Cecile Viboud, Wenqing Zhang, Laurence Torcel‐Pagnon, Cedric Mahe, Sandra S. Chaves

**Affiliations:** ^1^ Center of Excellence in Respiratory Pathogens (CERP), Hospices Civils de Lyon (HCL) and Centre International de Recherche en Infectiologie (CIRI), Équipe Santé Publique, Épidémiologie et Écologie Évolutive des Maladies Infectieuses (PHE3ID), Inserm U1111, CNRS UMR5308, ENS de Lyon Université Claude Bernard Lyon 1 (UCBL Lyon 1) Lyon France; ^2^ Molecular Biology/Microbiology Research Laboratory Universidade Federal do Paraná Curitiba Brazil; ^3^ FISABIO‐Public Health Valencia Spain; ^4^ Dalhousie University Halifax Canada; ^5^ National Institute of Health lslamadad Pakistan; ^6^ South African Medical Research Council, Vaccines & Infectious Diseases Analytics (VIDA) Research Unit, Faculty of Health Sciences University of the Witwatersrand Johannesburg South Africa; ^7^ HCL and CIRI, Inserm U1111, CNRS UMR5308, ENS de Lyon, UCBL Lyon 1 Lyon France; ^8^ Patan Academy of Health Sciences Patan Nepal; ^9^ National Reference Laboratory for Viral Respiratory Diseases, Virology Department INEI‐ANLIS Buenos Aires Argentina; ^10^ Center for Infectious Diseases Research American University of Beirut Beirut Lebanon; ^11^ Gamaleya National Research Center for Epidemiology and Microbiology Ministry of Health of Russian Federation Moscow Russia; ^12^ Institut National d'Hygiène Publique (INHP) Abidjan Côte d'Ivoire; ^13^ School of Public Health University of Hong Kong Hong Kong China; ^14^ Smorodintsev Research Institute of Influenza Saint Petersburg Russia; ^15^ Institut Pasteur of Dakar Dakar Senegal; ^16^ National Institute for Infectious Diseases “Prof. Dr. Matei Bals” Bucharest Romania; ^17^ Epidemiology, Immunization and Infection Control Department Hospital Pequeno Principe Curitiba Brazil; ^18^ Grupo de Investigación UNIMOL, Facultad de Medicina, Universidad de Cartagena Cartagena de Indias Colombia; ^19^ Sher‐i‐Kashmir Institute Srinagar India; ^20^ National Institute of Public Health Prague Czech Republic; ^21^ Clínica Internacional, Instituto de Medicina Tropical Universidad Nacional Mayor de San Marcos Lima Peru; ^22^ Université Paris Cité, Assistance Publique ‐ Hôpitaux de Paris (AP‐HP), CIC Vaccinologie Cochin Pasteur, Hôpital Cochin, Inserm, FCRIN, I REIVAC Paris France; ^23^ AP‐HP, CIC Vaccinologie Cochin Pasteur, Hospital Cochin Paris France; ^24^ The CIRN Serious Outcomes Surveillance (SOS) Network Halifax Canada; ^25^ Department of Epidemiology, Faculty of Medicine University of Novi Sad Novi Sad Serbia; ^26^ SI Kyiv City Center for Diseases Control and Prevention of the Ministry of Health of Ukraine Kyiv Ukraine; ^27^ Medical University of Warsaw Warsaw Poland; ^28^ Kenya Medical Research Institute (KEMRI) Nairobi Kenya; ^29^ HCL Lyon France; ^30^ Instituto Nacional de Ciencias Medicas y Nutricion Salvador Zubiran Mexico City Mexico; ^31^ Institut Pasteur de Tunis Tunis Tunisia; ^32^ Davila University of Medicine and Pharmacy Bucharest Romania; ^33^ Icahn School of Medicine at Mount Sinai New York New York USA; ^34^ Vaccine Institute Hacettepe University Ankara Türkiye; ^35^ Department of Infectious Diseases and Clinical Microbiology Hacettepe University School of Medicine Ankara Türkiye; ^36^ HCL and CIRI, Épidémiologie et Écologie Évolutive des Maladies Infectieuses (PHE3ID), Inserm U1111, CNRS UMR5308, ENS de Lyon, UCBL Lyon 1 Lyon France; ^37^ School of Public Health Fudan University Shanghai China; ^38^ Impact Healthcare Paris France; ^39^ Partnership for International Vaccine Initiatives, The Task Force for Global Health Decatur Georgia USA; ^40^ Worldwide Influenza Centre The Francis Crick Institute London UK; ^41^ Center for Vaccine Development and Global Health University of Maryland School of Medicine Baltimore Maryland USA; ^42^ Division of International Epidemiology and Population Studies, Fogarty International Center National Institutes of Health Bethesda Maryland USA; ^43^ Global Influenza Program WHO Geneva Switzerland; ^44^ Foundation for Influenza Epidemiology Fondation de France Paris France

**Keywords:** influenza, international, public–private partnerships, respiratory viruses, surveillance

## Abstract

Respiratory viruses represent a significant public health threat. There is the need for robust and coordinated surveillance to guide global health responses. Established in 2012, the Global Influenza Hospital Surveillance Network (GIHSN) addresses this need by collecting clinical and virological data on persons with acute respiratory illnesses across a network of hospitals worldwide. GIHSN utilizes a standardized patient enrolment and data collection protocol across its study sites. It leverages pre‐existing national infrastructures and expert collaborations to facilitate comprehensive data collection. This includes demographic, clinical, epidemiological, and virologic data, and whole genome sequencing (WGS) for a subset of viruses. Sequencing data are shared in the Global Initiative on Sharing All Influenza Data (GISAID). GIHSN uses financing and governance approaches centered around public–private partnerships. Over time, GIHSN has included more than 100 hospitals across 27 countries and enrolled more than 168,000 hospitalized patients, identifying 27,562 cases of influenza and 44,629 of other respiratory viruses. GIHSN has expanded beyond influenza to include other respiratory viruses, particularly since the COVID‐19 pandemic. In November 2023, GIHSN strengthened its global impact through a memorandum of understanding with the World Health Organization, aimed at enhancing collaborative efforts and data sharing for improved health responses. GIHSN exemplifies the value of integrating scientific research with public health initiatives through global collaboration and public–private partnerships governance. Future efforts should enhance the scalability of such models and ensure their sustainability through continued public and private support.

## 1 Introduction

Surveillance platforms to monitor respiratory viral illness are crucial to public health. Established in 2012, the Global Influenza Hospital Surveillance Network (GIHSN) is a multicountry collaboration of sentinel hospitals monitoring acute respiratory infections in hospitalized patients. The network has actively collected year‐round data from thousands of respiratory hospitalizations, integrating clinical and virologic information, including virus genome sequencing [1].

A pivotal aspect of this network is its reliance on pre‐existing national infrastructures and expertise. All GIHSN coordinating sites have extensive experience in hospital‐based surveillance. Some of these institutions are actively engaged in their country's national surveillance efforts, including the World Health Organization (WHO) National Influenza Centres (NICs), public health institutes, and academic centers. GIHSN collaborators recruit local hospitals that adopt standardized patient enrolment approaches, collecting uniform data on respiratory hospitalizations and linking specimens tested for respiratory viruses with epidemiological and clinical data, including clinical outcomes during hospital stays. A subset of viruses undergo whole genome sequencing (WGS), and the information is uploaded into the Global Initiative on Sharing All Influenza Data (GISAID) platform [2]. The GIHSN mission and procedures align with discussions of the pandemic accord, emphasizing the necessity for robust international cooperation and data‐sharing mechanisms in pandemic preparedness and response [3].

From its inception, the GIHSN has employed innovative financing and governance approaches centered around public–private partnerships (PPP) to effectively coordinate a sustainable surveillance initiative with global reach.

## 2 The GIHSN Over the Years

**FIGURE 1:**
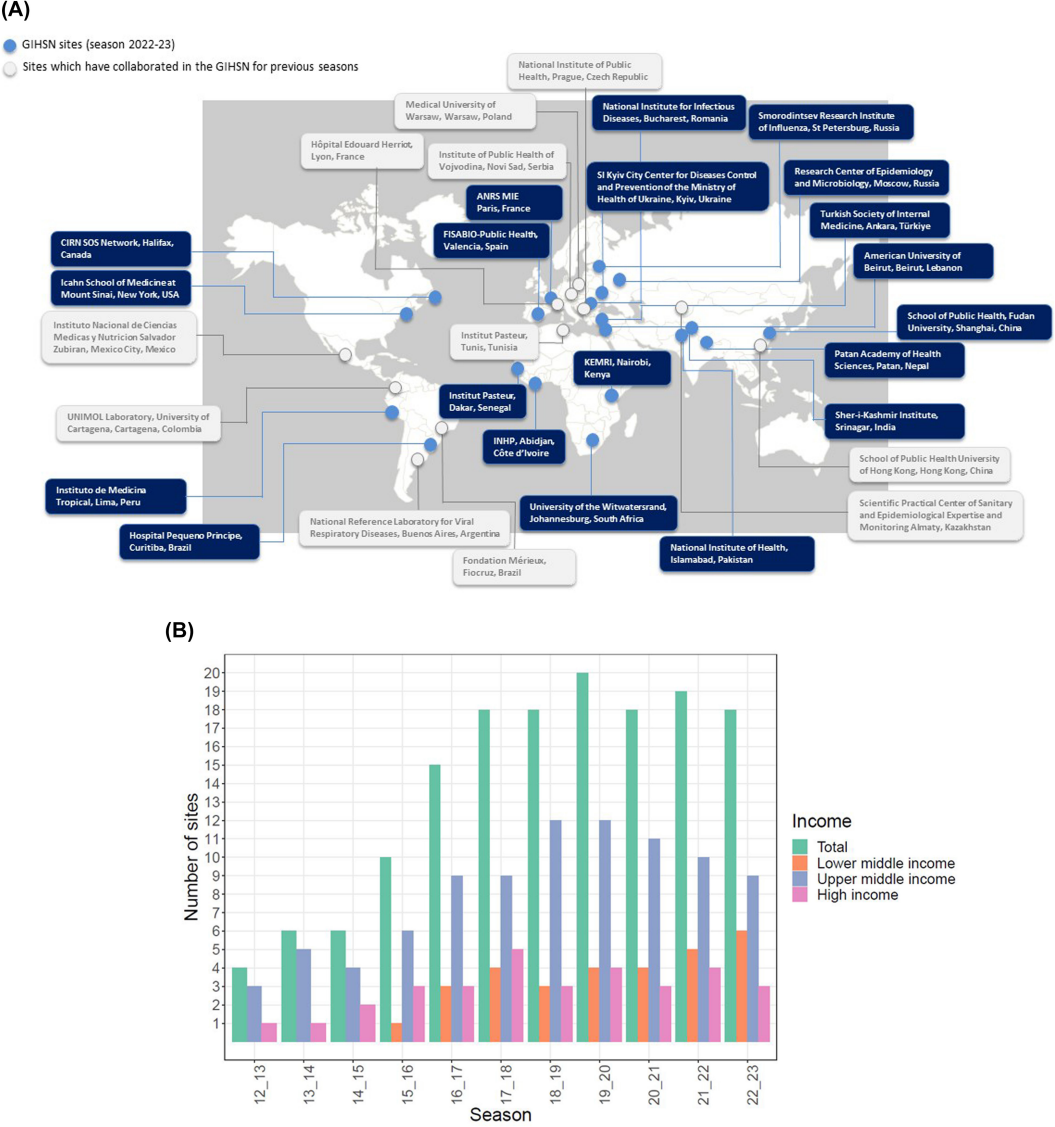
Distribution of the collaborating sites participating in the Global Influenza Hospital Surveillance Network. (A) Geographical distribution of the collaborating sites participating in the Global Influenza Hospital Surveillance Network. (B) Number of collaborating sites participating in the Global Influenza Hospital Surveillance Network per season.

For more than a decade, the GIHSN has contributed to the monitoring of influenza and other respiratory viruses. It has included more than 100 hospitals across 27 countries and five continents. Figure 1A illustrates the geographical distribution of the sites collaborating within the GIHSN, from the 2012–2013 season to 2022–2023.

In its inaugural season in 2012–2013, the network was composed of four coordinating sites [4, 5]. During the first three seasons, network sites were predominantly in the Northern Hemisphere and concentrated within upper‐middle or high‐income countries [4, 6, 7]. However, since 2015, there has been a notable and intentional increase in geographical representation, with sites spread across diverse geographical locations and an increase in the number from lower and middle‐income settings (Figure 1B) [8, 9], in an attempt to ensure the collection of data that are more representative of the populations at risk and the varying seasonality of respiratory viruses globally.

**FIGURE 2:**
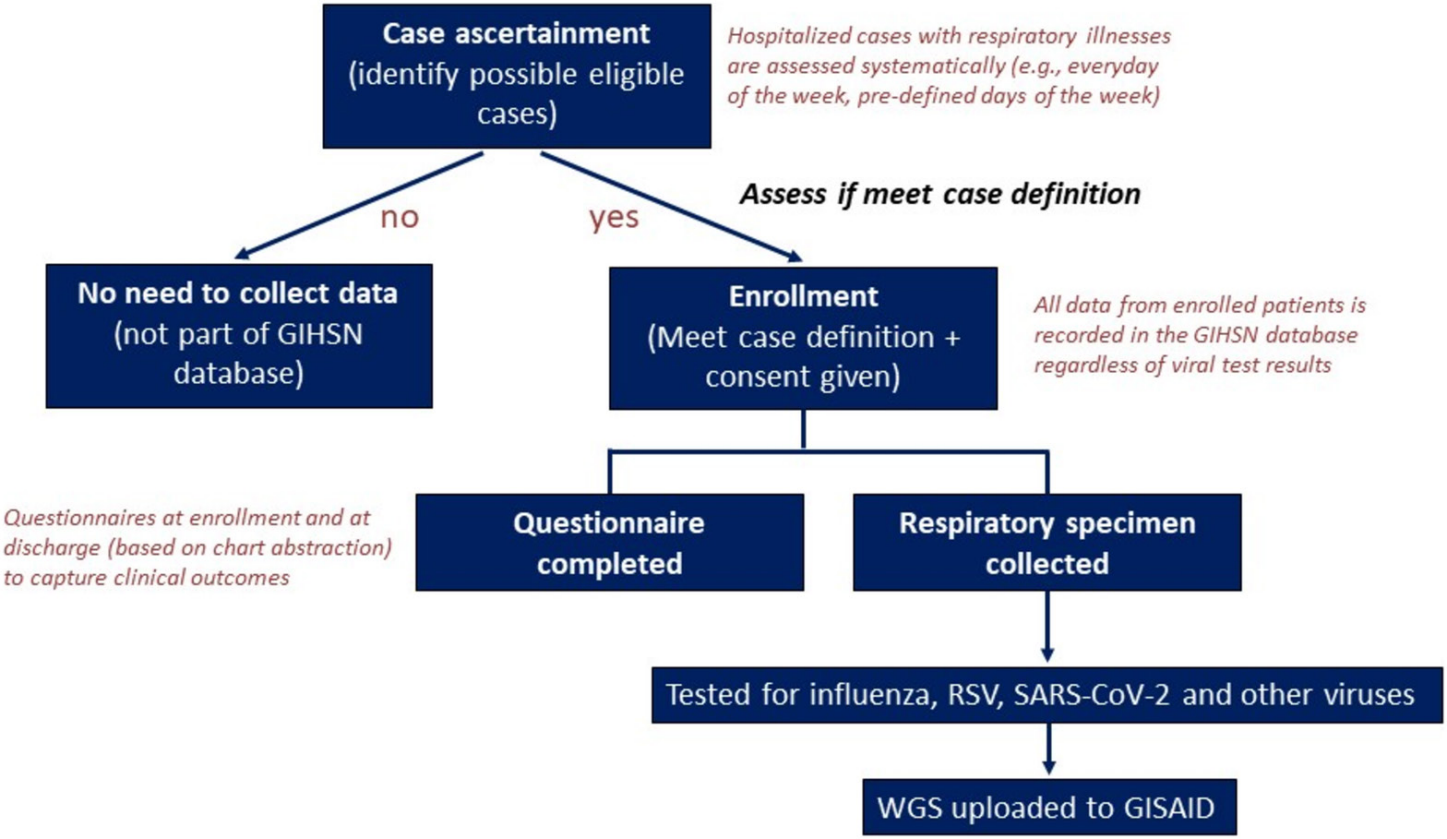
Patients' enrolment and data collection flow in the Global Influenza Hospital Surveillance Network. GIHSN: Global Influenza Hospital Surveillance Network; RSV: respiratory syncytial virus; SARS‐CoV‐2: severe acute respiratory syndrome coronavirus 2; WGS: whole genome sequencing; GISAID: Global Initiative on Sharing All Influenza Data.

**FIGURE 3:**
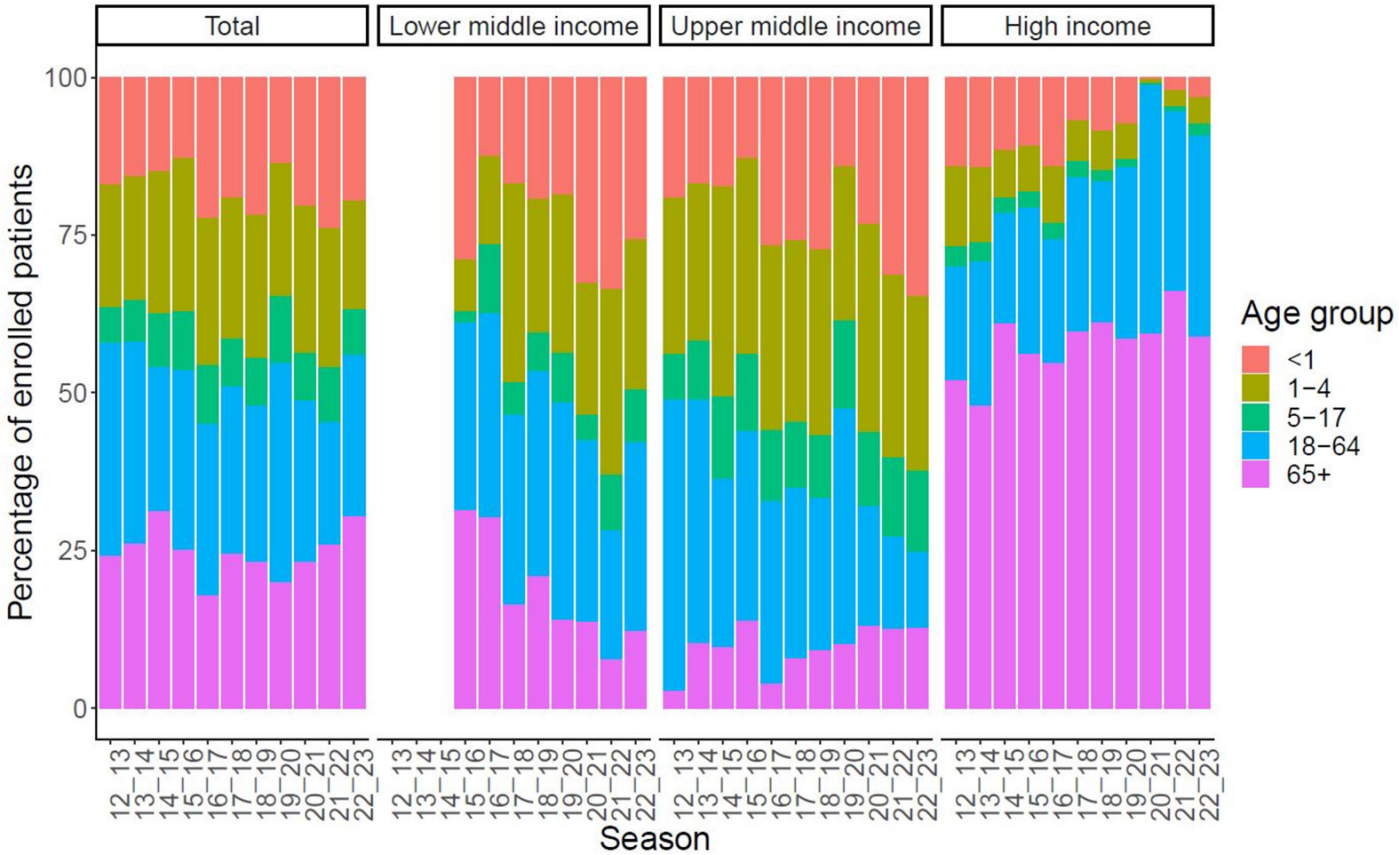
Distribution of enrolled patients in the Global Influenza Hospital Surveillance Network from 2012 to 2023 according to age and country income level.

The coordinating sites operate following a standardized core protocol and use a common questionnaire to capture patient data uniformly. Individual patient‐level demographic, clinical, epidemiological, and virologic information are systematically recorded from patients admitted with respiratory illnesses who meet predetermined eligibility criteria (Figure 2). Importantly, information is collected on the continuum of illness, from prehospitalization signs, symptoms, and management to hospital documented disease severity, as well as treatment and clinical outcomes and characterization of the patients' comorbidity profile. Data management systems were implemented to ensure data quality and comparability, enabling efficient data pooling among sites. Whereas hospitalization patterns vary by age and setting, the overall global surveillance platform includes pediatric and adult hospitals, as well as specialized and general hospitals, allowing a wide representation of patients of all ages to be captured (Figure 3).

Virus testing is conducted using polymerase chain reaction (PCR); for several sites, the GIHSN was catalytic to improving their surveillance and diagnosis standards over the years. The GIHSN has further expanded the scope of surveillance to encompass genomic sequencing. As such, sites submit sequencing data from a subset of their positive specimens, preferably WGS, or alternatively the influenza hemagglutinin (HA) and neuraminidase (NA) next‐generation consensus sequences to the EpiFlu database of GISAID [2]. To facilitate the introduction of sequencing into local influenza surveillance, the GIHSN has developed and provided a sequencing protocol that helped set‐up the process in several sites. For sites lacking independent sequencing capabilities, there is a provision to send samples to the NIC's sequencing platform in Lyon, France, or to the WHO Collaborating Centres to ensure that all sites can contribute viral genome data without limitations imposed by limited resources.

The GIHSN has evolved into an integrated surveillance and scientific collaboration, adapting to meet changing public health needs and objectives. While its overarching goal to delineate the epidemiology of viral‐associated hospitalizations and gain insights into viral strain circulation, severity, and risk factors remains constant, other objectives have evolved in response to public health needs. For example, during the coronavirus disease 2019 (COVID‐19) pandemic, when disruptions in virus circulation were documented globally [10], it became clear that monitoring virus circulation year‐round was needed. Consequently, GIHSN expanded its focus from primarily tracking influenza and other respiratory viruses during the respiratory seasons to encompassing continuous data collection on a broader range of respiratory viruses (Table 1). This shift was facilitated by the widespread adoption of multiplex PCR techniques, enabling the identification of multiple viruses. Notably, since 2019, enhancements also included integrating epidemiological and clinical information with viral WGS data. This integration aims to investigate the relationship between viral genomic changes and disease severity, including vaccine breakthrough cases, thereby aiding in vaccine strain selection [11]. Whole genome sequencing and severity data are promptly shared with the WHO to inform vaccine composition decisions in their twice‐yearly meetings.

**TABLE 1:** Viruses tested by the different collaborating sites during the 2022–2023 season.

Country	Coordinating site	Influenza	SARS‐CoV‐2	RSV	HCoV	HMPV	AdV	HBoV	HPIV	RhV	Other viruses
**High‐income**											
Canada	CIRN Serious Outcomes Surveillance Network, Halifax	X	X	X		X			X	X	X
Spain	FISABIO‐Public Health, Valencia	X	X	X	X	X	X	X	X	X	X
United States	Icahn School of Medicine at Mount Sinai, New York	X	X	X	X	X	X		X	X	X
**Upper‐middle income**											
Brazil	Hospital Pequeno Principe, Curitiba	X	X	X	X	X	X	X	X	X	X
Lebanon	American University of Beirut, Beirut	X	X	X	X	X	X	X	X	X	X
Peru	Instituto de Medicina Tropical, Lima	X	X	X		X	X				
Romania	National Institute for Infectious Diseases, Bucharest	X	X	X	X	X	X	X	X	X	X
Russia	Gamaleya National Research Center for Epidemiology and Microbiology	X	X	X	X	X	X	X	X	X	X
Russia	Smorodintsev Research Institute of Influenza, St. Petersburg	X	Х	X	X	Х	Х	X	Х	X	
South Africa	University of the Witwatersrand Johannesburg	X	X	X		X	X		X	X	
Türkiye	Turkish Society of Internal Medicine, Ankara	X	X	X	X	X	X	X	X	X	X
Ukraine	SI Kyiv City Center for Diseases Control and Prevention of the Ministry of Health of Ukraine, Kyiv	X	X								
**Lower‐middle income**											
India	Sher‐i‐Kashmir Institute, Srinagar	X									
Côte d'Ivoire	Institut National d'Hygiène Publique, Abidjan	X	X	X	X	X					
Kenya	Kenya Medical Research Institute, Nairobi	X	X								
Nepal	Patan Academy of Health Sciences, Patan	X	X								
Pakistan	National Institute of Health, lslamabad	X	X	X	X	X	X		X	X	
Senegal	Institut Pasteur of Dakar, Dakar	X	X	X	X	X	X	X	X	X	X

Abbreviations: AdV: adenovirus; HBoV: human bocavirus; hCoV: human coronaviruses; HMPV: human metapneumovirus; HPIV: human parainfluenza viruses; RhV: rhinovirus; RSV: respiratory syncytial virus.

## 3 The GIHSN Resilience

As of October 2023, GIHSN enrolled 168,639 hospitalized patients, identifying 27,562 cases of influenza and 44,629 of other respiratory viral illnesses.

The GIHSN has proved to be a collective effort that has withstood social, environmental, political, and health challenges; relying on an empowered community of researchers from civil society makes it less sensitive to political agendas. Over time, some of the challenges encountered, like the COVID‐19 pandemic, affected all sites. Other natural and human‐made disasters have affected specific sites or regions in unique ways—such as sites that have been affected by earthquakes, flooding, and rolling power outages affecting the stability of internet connections. Some contributing countries have experienced conflict and war, but their participation has been continuous despite enormous upheaval and challenges. The COVID‐19 pandemic presented immense challenges, but also a unique opportunity for GIHSN, to step up and contribute to the pandemic response [12]. This readiness to adapt and act highlights the importance of continuously supporting surveillance and research infrastructures so that they are in place and ready to be adapted to address novel and emerging threats. From the onset of the COVID‐19 pandemic, the coordinating sites employed the network's capabilities for collecting data on SARS‐CoV‐2. Many sites monitored SARS‐CoV‐2 activity by integrating it into their existing influenza surveillance systems while continuing their influenza surveillance efforts.

**FIGURE 4:**
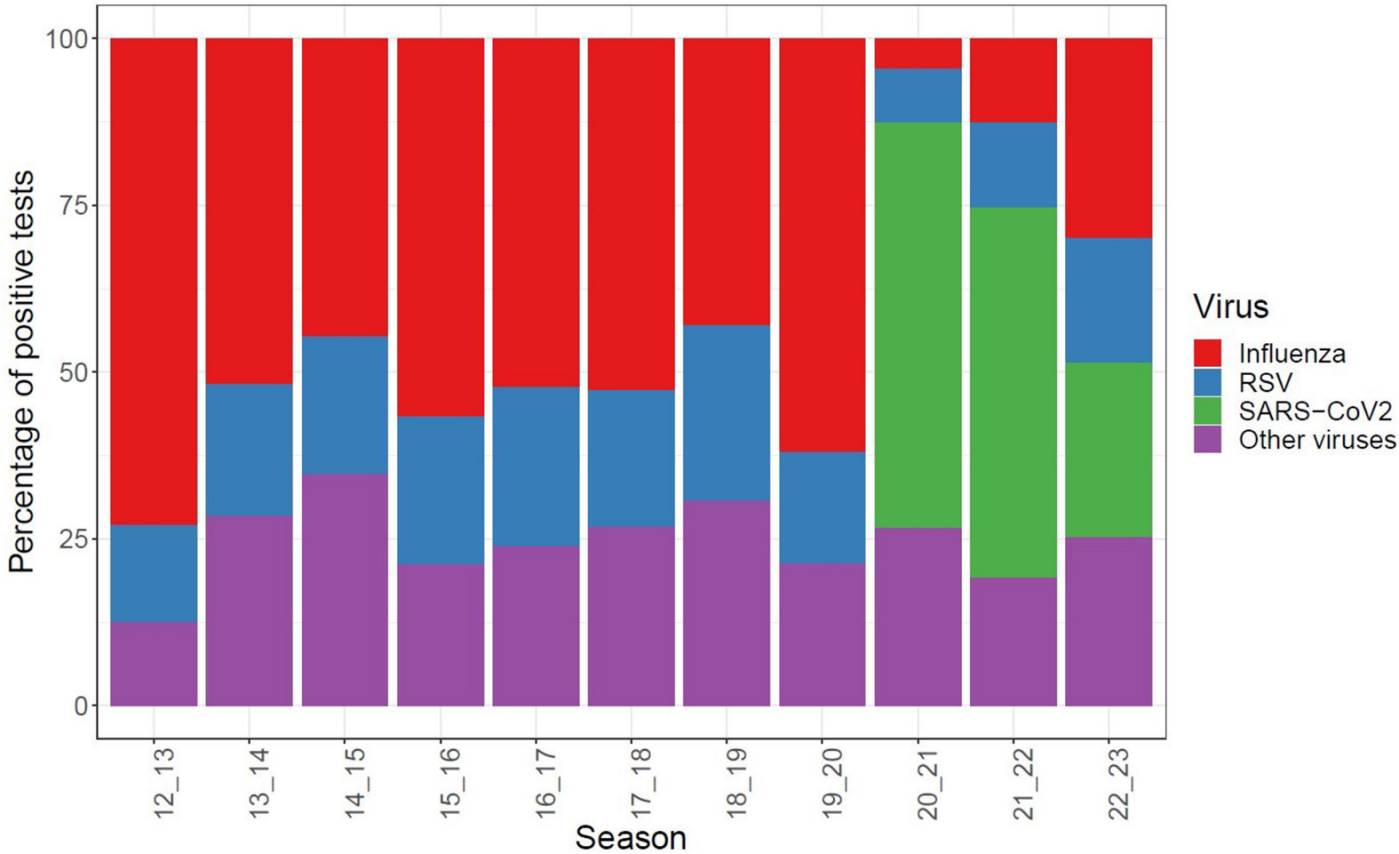
Distribution of detected viruses from 2012 to 2023 in the enrolled patients in the Global Influenza Hospital Surveillance Network. RSV: respiratory syncytial virus; SARS‐CoV‐2: severe acute respiratory syndrome coronavirus 2.

The emergence of SARS‐CoV‐2 had a profound impact on the circulation of influenza and other respiratory viruses. Although the total number of patients enrolled by GIHSN sites during the 2019–2020 and 2020–2021 seasons (> 14,300 in each season) remained similar to previous years and increased for the 2021–2022 season (> 25,500), there was a substantial decrease in the frequency of influenza detections (Figure 4). In most temperate Northern Hemisphere countries, the 2019–2020 influenza epidemic was concluding as cases of SARS‐CoV‐2 began to surge in March 2020. Consequently, in these regions, few to no new influenza cases were reported during the typical influenza season (between December and February) during 2020–2021. In accordance with many reports, the GIHSN findings underscore how the timing and intensity of influenza activity deviated from the usual and were often limited between 2020 and 2022 [10]. During these two seasons, SARS‐CoV‐2 was detected in more than 70% of the patients with a positive viral result. Notably, the GISHN sites were also among the sources reporting no circulation of influenza B‐Yamagata lineage from 2021 [13].

## 4 The GIHSN Governance

Since its establishment, the GIHSN has leveraged innovative financing through a PPP governance model. With the COVID‐19 pandemic demonstrating that partnerships could lead to more agile networks, co‐construction by both the public and private sectors holds great relevance for the success of infectious disease surveillance systems given the importance of the effort, the multisectoral relevance, and the scarce resources available. PPP offers structural flexibility, free from the constraints that independent public‐sector, private‐sector philanthropic, and civil society entities often face when pursuing new initiatives [14, 15].

**FIGURE 5:**
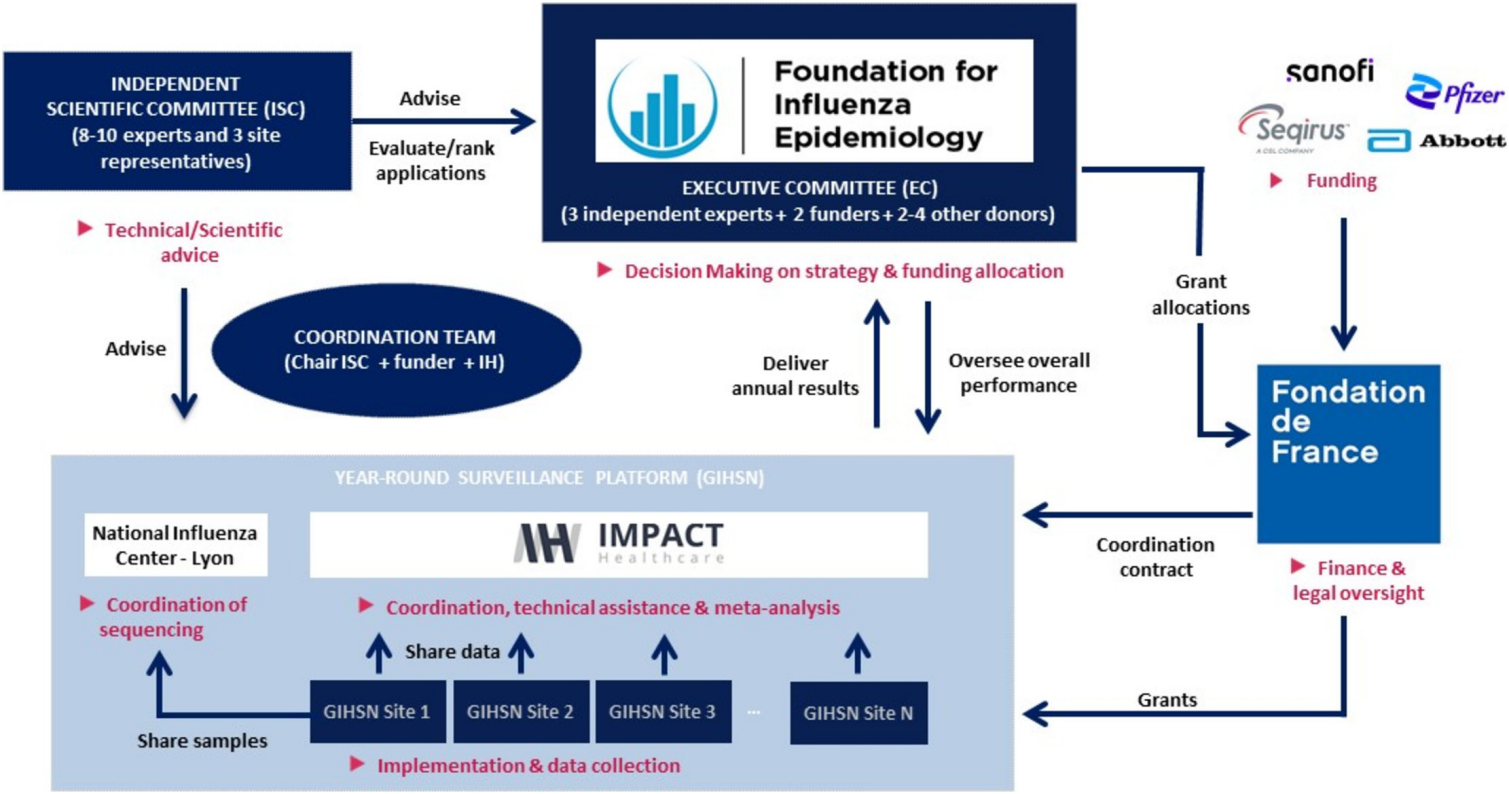
Governance and operationalization of the Global Influenza Hospital Surveillance Network.

The GIHSN is supported by a dedicated fund hosted by the *Fondation de France*, a not‐for‐profit private institution set up by the French government. This fund, named the Foundation for Influenza Epidemiology (FIE), was created in September 2015 by Sanofi to formalize several years of commitment to epidemiological research on influenza [16]. The FIE provides catalytic funding to the coordinating sites under a yearly grant to enhance surveillance and research capacity. This funding represents, on average, about 25% of the actual cost of the surveillance because it relies mainly on existing national capacity and infrastructure (co‐funding from Ministries of Health for many sites, or other funding agencies such as the Bill & Melinda Gates Foundation, US‐CDC cooperative agreements and WHO resources), resulting in bidirectional leveraging of research opportunities and data sharing. To date, the FIE has allocated over €17 millions to develop and expand the GIHSN platform. The FIE budget consists of unrestricted grants from the private sector, with contributions from Sanofi, Seqirus (from 2020), Abbott Diagnostics (from 2022), the International Federation of Pharmaceutical Manufacturers and Associations (IFPMA) (from 2019 to 2020), Illumina (from 2021 to 2022), and Pfizer since early 2024 (Figure 5). Donors do not have direct access to the database, and all results are made publicly available through sharing of data during the annual GIHSN meeting, conferences, and peer‐reviewed publications [1].

The FIE's governance is upheld through an Executive Committee responsible for charting GIHSN strategic course. Operating under established criteria, which are detailed in the GIHSN's public calls and guided by recommendations from the Independent Scientific Committee (ISC), the Executive Committee annually selects study sites. The Executive Committee comprises representatives of the donors and independent experts who are part of the ISC. The ISC is composed of eminent experts in epidemiology, virology, and public health, as well as investigators from the network sites. The ISC plays a crucial role in ensuring the GIHSN's scientific oversight. The coordination of the network, the oversight of study implementation, and the management and hosting of the data are supported by Impact Healthcare, an independent research organization headquartered in Paris, France [17].

To comply with data access and privacy regulations, the FIE has established a data warehouse alongside a comprehensive data access framework. This setup guarantees that the GIHSN database is securely maintained, with data processing strictly conforming to the European General Data Protection Regulation and French data protection laws. Before field implementation starts, each GIHSN site signs a data‐sharing agreement stating compliance with both ethical and national regulations for surveillance activities. Importantly, all data collected by the sites under the surveillance protocol remains the site's proprietary. Sites are then invited to participate in proposed analyses and are free to decline to contribute their data should they so choose.

Since 2021, FIE has invested part of its budget in supporting research projects and analytical proposals on influenza and other respiratory viruses that leverage the GIHSN platform and database, considering the volume of data collected. The network sites or other not‐for‐profit institutions are welcome to submit proposals that include either novel analyses of existing GIHSN data, or the use of respiratory specimens for pathogen discovery or other relevant studies. Site‐specific investigators can also be engaged directly, to collect new data to further our understanding of influenza and other respiratory viruses and related vaccines.

## 5 Scientific Highlights

Each year, under the supervision of the ISC, at least one scientific manuscript is published combining data from the various GIHSN sites. These manuscripts describe different aspects of each season's findings [4, 5, 6, 7, 8, 9, 11, 18, 19, 20, 21]. Additionally, individual sites are encouraged to publish their specific data [22, 23, 24, 25, 26, 27, 28, 29, 30, 31, 32, 33].

Any additional analyses aligned with the GIHSN scope are conducted following a thorough review and approval process by both the ISC and the FIE. Notably, study sites are proactively informed and associated with these analyses, and they are given the option to opt‐out. As an example, one recent analysis delved into assessing the risk associated with intensive care unit admissions, mechanical ventilation, and in‐hospital death among hospitalized influenza patients. This analysis explored the influence of patient‐level covariates and country income, shedding light on critical aspects of influenza‐related healthcare outcomes [19].

Moreover, the GIHSN platform offers a distinctive opportunity for the advancement of pathogen discovery initiatives. For instance, the availability of archived respiratory samples proved useful in ascertaining when SARS‐CoV‐2 started to circulate in the Valencia region in Spain [29]. More recently, the US‐CDC has been exploring the possibility of a collaborative effort to establish surveillance for enterovirus D68 at specific GIHSN sites.

The GIHSN has also contributed to initiatives evaluating the burden of respiratory diseases, such as the Burden of Influenza and RSV Disease (BIRD) project [34, 35, 36].

## 6 Moving Forward

In an era marked by greater globalization, virus circulation between countries and regions has increased. This global connectivity also presents a unique opportunity for collaborative public health research on respiratory infections, necessitating a shift from solely local to a more comprehensive global analysis. The GIHSN, with its presence across the globe, offers an important perspective. Its alignment with current discussions on enhancing pandemic preparedness makes it an ideal platform for fostering synergistic collaborations and shared initiatives [15].

The emergence of the COVID‐19 pandemic has spurred significant investments and attention toward surveillance infrastructure, resulting in tangible improvements in national surveillance capabilities. Integrating SARS‐CoV‐2 surveillance into existing influenza sentinel surveillance systems has paved the way for continued global monitoring of respiratory viruses with pandemic potential. However, despite the universally acknowledged value of such surveillance networks, ensuring their long‐term financial sustainability remains an ongoing challenge [37, 38]. For the improvements to be sustainable, further investments and steadfast commitments are imperative [15, 39]. PPP with robust governance structures, combined with the integration of data standards such as those championed by GIHSN, will play a pivotal role in ensuring this long‐term sustainability. The expanding circle of GIHSN funders, now including more entities from the vaccine and diagnostic sectors, underscores a growing industry commitment to bolster public health surveillance. This collective approach, incorporating co‐financing and blended financing models, offers a viable pathway to enhance and diversify support for global health surveillance networks [40].

The extensive GIHSN database, encompassing data from over 168,639 acute respiratory hospitalized patients worldwide, is valuable for investigating epidemiological questions that transcend the network's original objectives. The growing availability of information on influenza virus gene sequences enhances our understanding of the molecular dynamics and epidemiology of these viruses. With the scale‐up of genome sequencing, the use of GIHSN data should evolve to not only supplement WHO networks with sequence data submissions to the GISAID platform but also to incorporate detailed reports on disease severity. This enriched dataset can provide a more nuanced understanding of influenza breakthrough infections and strengthening of global pandemic readiness. Global surveillance on other respiratory viruses such as respiratory syncytial virus, including collection of clinical data and the impact of immunization strategies will be of increasing importance in the short term, and the GIHSN is poised to contribute important data to public health policy decisions.

Recognizing the need for enhanced collaboration in an evolving public health landscape, GIHSN has sought to strengthen partnerships and synergies, notably with the Global Influenza Surveillance & Response System (GISRS), a cornerstone of influenza laboratory testing supported by the WHO for over 70 years [41]. In November 2023, a memorandum of understanding was signed between the WHO and the *Fondation de France*, allowing for more formal collaborations, aiming to complement the GISRS network and to amplify data communication to both national and international health organizations. This initiative underscores GIHSN's commitment to improving respiratory pathogen surveillance and informing decision‐makers, emphasizing the critical importance of prompt, high‐quality data collection, sharing, and transparency for the benefit of the global community.

Moreover, GIHSN's approach is aligned with the innovative WHO Mosaic surveillance framework, which proposes a tripartite domain structure for surveillance [39]. This framework underscores the necessity for integrated, purpose‐built surveillance systems tailored to meet the specific objectives of tracking respiratory viruses with the potential to cause epidemics and pandemics [39]. Although some surveillance platforms may have robust geographic coverage, they often lack the granularity to understand disease nuances by risk groups or severity thoroughly [42, 43, 44, 45, 46]. The GIHSN addresses this gap by employing a standard protocol and collecting detailed clinical outcome information, thereby enriching the overall characterization of diseases. This approach not only meets current public health needs but is also adaptable to evolving requirements, standing as a testament to the power of collaborative, multifaceted surveillance strategies in addressing pressing public health challenges and adding critical pieces to the epidemiological puzzle.

## 7 Conclusion

The GIHSN exemplifies a pivotal model for global surveillance, demonstrating scalability and flexibility. Through adopting a common protocol and implementing active, prospective surveillance across numerous countries, the GIHSN harnesses large sample sizes and provides extensive global virologic and clinical data related to severe respiratory illnesses throughout the year. Notably, this initiative has already garnered the involvement of various private partners. The global landscape of disease surveillance could benefit from approaches like the GIHSN, as multisector partnerships offer a range of values that extend beyond what can be achieved through collaboration solely among governments or other entities.

Scientific instruments like GIHSN are grounded on pragmatism and easily scalable, pending availability of resources. Importantly, they prioritize empowering the network of participating sites, fostering a “glocal” (having properties relevant for both local and global levels) model that represents a departure from top‐down approaches, emphasizing adaptability and local engagement. The network's ability in refining its objectives based on evolving external circumstances and emerging needs, such as the expansion of genome sequencing or broader focus on respiratory pathogens, underscores its agility.

In summary, the GIHSN stands as a catalyst for uniting diverse stakeholders around a timely and adaptable platform capable of addressing critical data requirements in both pandemic and interpandemic times. The importance of sustainable surveillance networks, underpinned by collaborative efforts, cannot be overstated in their role in supporting and complementing existing public health strategies.

## Author Contributions

Conceptualization: Marta C. Nunes, Joseph Bresee, Bruno Lina, John W. McCauley, Justin R. Ortiz, Cecile Viboud, Wenqing Zhang, Laurence Torcel‐Pagnon, Cedric Mahe, and Sandra S Chaves. Methodology: Marta C. Nunes, and Sandra S Chaves. Data curation: Catherine Commaille‐Chapus, and Camille Hunsinger. Investigation: Antonin Bal, Gregory Destras, Laurence Josset, Hadrien Regue, and Bruno Lina. Formal analysis: Cecile Chauvel. Supervision: Marta C. Nunes, Sonia M. Raboni, F. Xavier López‐Labrador, Melissa K. Andrew, Nazish Badar, Vicky Baillie, Kedar Baral, Elsa Baumeister, Celina Boutros, Elena Burtseva, Daouda Coulibaly, Ben Cowling, Daria Danilenko, Ghassan Dbaibo, Ndongo Dia, Anca Cristina Drăgănescu, Heloisa I. G. Giamberardino, Doris Gomez‐Camargo, Parvaiz A. Koul, Jan Kyncl, Victor Alberto Laguna‐Torres, Odile Launay, Liem Binh Luong Nugyen, Shelly McNeil, Snežana Me, Ainara Mira‐Iglesias, Alla Mironenko, Aneta Nitsch‐Osuch, Alejandro Orrico‐Sánchez, Nancy A. Otieno, Guillermo M. Ruiz‐Palacios, Afif Ben Salah, Muhammad Salman, Oana Săndulescu, Viviana Simon, Anna Sominina, Emilia Sordillo, Mine Durusu Tanriover, Serhat Unal, Harm van Bakel, Philippe Vanhems, Tao Zhang, Catherine Commaille‐Chapus, Laurence Torcel‐Pagnon, and Cedric Mahe. Visualization: Cecile Chauvel. Project administration: Sonia M. Raboni, F. Xavier López‐Labrador, Melissa K. Andrew, Nazish Badar, Vicky Baillie, Kedar Baral, Elsa Baumeister, Celina Boutros, Elena Burtseva, Daouda Coulibaly, Ben Cowling, Daria Danilenko, Ghassan Dbaibo, Ndongo Dia, Anca Cristina Drăgănescu, Heloisa I. G. Giamberardino, Doris Gomez‐Camargo, Parvaiz A. Koul, Jan Kyncl, Victor Alberto Laguna‐Torres, Odile Launay, Liem Binh Luong Nugyen, Shelly McNeil, Snežana Me, Ainara Mira‐Iglesias, Alla Mironenko, Aneta Nitsch‐Osuch, Alejandro Orrico‐Sánchez, Nancy A. Otieno, Guillermo M. Ruiz‐Palacios, Afif Ben Salah, Muhammad Salman, Oana Săndulescu, Viviana Simon, Anna Sominina, Emilia Sordillo, Mine Durusu Tanriover, Serhat Unal, Harm van Bakel, Philippe Vanhems, and Tao Zhang. Writing – original draft: Marta C. Nunes. Writing – review & editing: Marta C. Nunes, Cecile Chauvel, Sonia M. Raboni, F. Xavier López‐Labrador, Melissa K. Andrew, Nazish Badar, Vicky Baillie, Antonin Bal, Kedar Baral, Elsa Baumeister, Celina Boutros, Elena Burtseva, Daouda Coulibaly, Ben Cowling, Daria Danilenko, Ghassan Dbaibo, Gregory Destras, Ndongo Dia, Anca Cristina Drăgănescu, Heloisa I. G. Giamberardino, Doris Gomez‐Camargo, Laurence Josset, Parvaiz A. Koul, Jan Kyncl, Victor Alberto Laguna‐Torres, Odile Launay, Liem Binh Luong Nugyen, Shelly McNeil, Snežana Me, Ainara Mira‐Iglesias, Alla Mironenko, Aneta Nitsch‐Osuch, Alejandro Orrico‐Sánchez, Nancy A. Otieno, Hadrien Regue, Guillermo M. Ruiz‐Palacios, Afif Ben Salah, Muhammad Salman, Oana Săndulescu, Viviana Simon, Anna Sominina, Emilia Sordillo, Mine Durusu Tanriover, Serhat Unal, Harm van Bakel, Philippe Vanhems, Tao Zhang, Catherine Commaille‐Chapus, Camille Hunsinger, Joseph Bresee, Bruno Lina, John W. McCauley, Justin R. Ortiz, Cecile Viboud, Wenqing Zhang, Laurence Torcel‐Pagnon, Cedric Mahe, and Sandra S Chaves.

## Conflicts of Interest

LT‐P, CM, and SSC work for Sanofi when not seconded to the Foundation for Influenza Epidemiology.

### Peer Review

The peer review history for this article is available at https://www.webofscience.com/api/gateway/wos/peer‐review/10.1111/irv.70091.

## Data Availability

Anonymized data are available on request to contact@gihsn.org. The use of data depends on the approval of an analytical proposal by the Independent Scientific Committee. Investigators from participant sites are informed up front for any planned data analysis, and they have the possibility to opt out.
